# Association between different hemoglobin glycation index and prognosis in patients with a first diagnosis of acute myocardial infarction: a retrospective study based on the MIMIC-IV database

**DOI:** 10.3389/fcvm.2025.1447420

**Published:** 2025-05-26

**Authors:** Dong Chen, Ben Hu, Xing-hua Chen, Xing Wei, Jun Feng, Ze-ping Hu

**Affiliations:** ^1^ Department of Cardiology, The First Affiliated Hospital, Anhui Medical University, Hefei, Anhui, China; ^2^ Department of Cardiology, The Second People’s Hospital of Hefei, Hefei Hospital Affiliated to Anhui Medical University, Hefei, Anhui, China; ^3^ Intensive Care Unit, The Second People’s Hospital of Hefei, Hefei Hospital Affiliated to Anhui Medical University, Hefei, China

**Keywords:** acute myocardial infarction, hemoglobin glycation index, mortality, prognosis, MIMIC-IV

## Abstract

**Background:**

The hemoglobin glycation index (HGI) is defined as the difference between the observed and predicted values of glycosylated hemoglobin (HbA1c), which is closely associated with a variety of poor prognoses. However, the relationship between HGI and short-term mortality risk in patients with a first diagnosis of acute myocardial infarction (AMI) remains unclear. This study aims to provide a better understanding of the relationship between HGI and mortality risk in patients with a first diagnosis of AMI.

**Methods:**

We conducted a cohort study using data from 1,961 patients with a first diagnosis of AMI from the Medical Information Mart for Intensive Care IV (MIMIC-IV; version 2.2) database. Patients were divided into four groups based on HGI quartiles. A Cox proportional hazards model and a two-segmented Cox proportional hazards model were used to elucidate the non-linear relationship between HGI in patients with a first diagnosis of AMI and mortality.

**Results:**

Of the surveyed population, 175 patients (8.92%) died within 90 days, and 210 patients (10.71%) died within 180 days. A low HGI was significantly associated with 90-day mortality [HR, 1.99; 95% CI (1.22, 3.08); *P* < 0.001] and 180-day mortality [HR, 1.74; 95% CI (1.18, 2.43); *P* < 0.001] in patients with a first diagnosis of AMI in the completely adjusted Cox proportional risk model, showing a non-linear correlation with an inflection point at 0.16 and 0.44. In the subgroup analysis, patients with prediabetes mellitus (pre-DM) and lower HGI levels had increased 90-day [HR, 8.30; 95% CI (2.91, 23.68)] and 180-day mortality risks [HR, 6.84; 95% CI (2.86, 16.34)].

**Conclusion:**

There is a significant correlation between HGI and all-cause mortality in patients diagnosed with AMI, especially those with lower HGI. HGI can serve as a potential indicator for evaluating the 90 and 180-day death risk of such patients.

## 1 Introduction

As the global population ages and expands, acute myocardial infarction (AMI) has become the leading cause of death globally (1), killing more than 2 million people in the United States each year (2). With the standardization of pharmacological treatments and the development of coronary interventions, the mortality rate of AMI has fallen dramatically (3). However, an increasing number of patients have adverse outcomes that may be exacerbated by metabolic disorders (4), and the prognostic management of patients with AMI is a current medical priority that requires attention.

Stable blood glucose levels have long-term benefits for AMI patients (5). Glycated hemoglobin (HbA1c) is used in the diagnosis and treatment of individuals with diabetes, reflects an individual's average blood glucose level over a 3-month period, and is currently the most commonly used surrogate for the effectiveness of glucose-lowering interventions (6, 7). However, some studies have suggested that erythrocyte renewal and glucose gradients across the erythrocyte membrane may influence HbA1c levels, and in addition, mean erythrocyte lifespan, differences in cell membrane glucose transmembrane gradients, enzyme abnormalities, and genetic factors have independent effects on HbA1c (8). Therefore, HbA1c measurements do not fully reflect the state of glycemic metabolism. Hempe et al. developed the hemoglobin glycation index (HGI) to quantify the relationship between HbA1c and plasma glucose concentration (7, 9). HGI is defined as the difference between observed and predicted HbA1c in a linear regression equation fitted to fasting plasma glucose (FPG), which can be easily used in clinical research to avoid fluctuations of HbA1c (10).

Several studies have shown that both low and high HGI are strongly associated with an increased risk of atherosclerosis (11–13). In different populations, different HGI values reflect individual differences in glucose metabolism and Hb glycosylation. HGI is related to HbA1c and FPG, which reflect long-term and short-term glycaemic control, and can be a relatively intuitive reflection of a patient's glycaemic variability (7). Enhanced management of patients' HGI may improve their glycemic control. However, the relationship between HGI and prognosis in AMI patients has not been reported yet. Exploring the relationship between HGI and the prognosis of AMI patients will help us understand the relationship between glycemic metabolic status and the survival of AMI patients and identify high-risk patients. Therefore, in this study, we used the MIMIC-IV (version 2.2) database to construct linear regression equations to calculate HGI in patients with AMI and to analyze the correlation between HGI and adverse outcomes.

## 2 Method

### 2.1 Data source and study population

**Figure 1:**
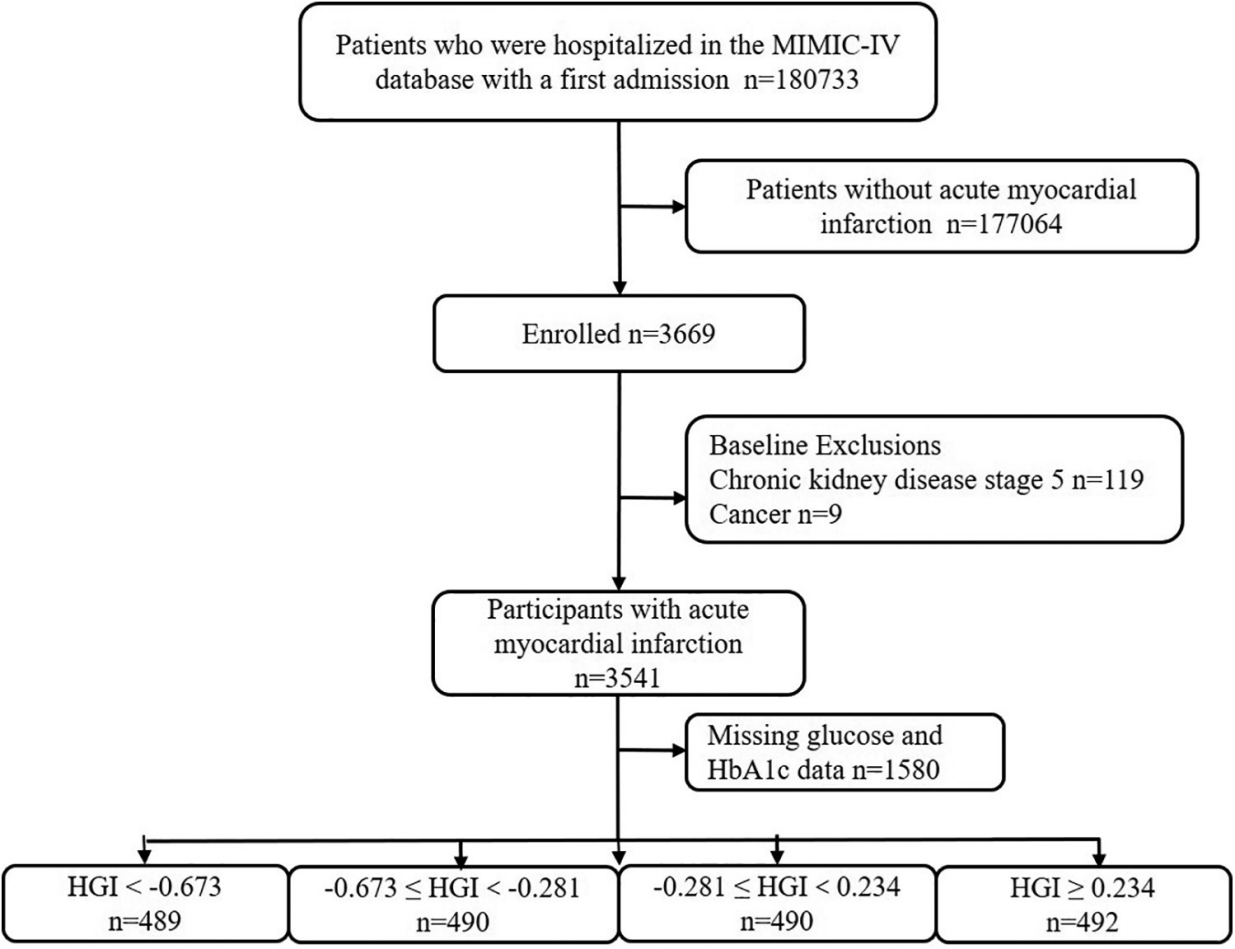
Flow diagram of study.

This retrospective study utilized health-related data from the MIMIC-IV (version 2.2) database, a comprehensive large-scale database developed and managed by the MIT Computational Physiology Laboratory. The database comprises over 50,000 high-quality medical records of patients admitted to the intensive care unit (ICU) at the Beth Israel Deaconess Medical Center (14). Notably, all personal identifying information has been anonymized to safeguard patient privacy. In light of the nature of this study, the Institutional Review Board of the Beth Israel Deaconess Medical Center waived the requirement for informed consent (14). To access the database, the author (XC) obtained the necessary certification and then extracted the required variables (Certification No.: 58951192). For this study, patients with a first diagnosis of AMI based on the International Classification of Diseases (ICD), ICD-9, and ICD-10 were included. For improving the accuracy and extrapolation of studies, participants under the age of 18 years (*n* = 0), those with chronic kidney disease (CKD) stage 5 (*n* = 119), with malignant tumors (*n* = 9), and those with missing glucose and HbA1c data (*n* = 1,580) were excluded. In the final analysis, 1,961 individuals were included and categorized into four groups based on the quartiles of their HGI levels (Figure 1).

### 2.2 Data collection

Data extraction was performed using the PostgreSQL tool (v.14, PostgreSQL Global Development Group, Berkeley, CA, USA). Diagnoses of patients were retrieved as per ICD-9 and ICD-10 using structured query language (SQL). Baseline characteristics, laboratory indicators, and medication usage records were queried through the item IDs stored in MIMIC-IV and matched to selected patients using a unique identifier (subject_id). Extracted variables included the following: (1) baseline characteristics, such as age, sex, height, weight, systemic inflammatory response (SIRS), sequential organ failure assessment (SOFA) (15); (2) comorbidities, such as hypertension, cardiogenic shock, atrial fibrillation, cardiac arrest, heart failure, diabetes, chronic kidney disease stage 5, malignant tumor (16); (3) laboratory parameters, such as red blood cell (RBC), white blood cell (WBC), platelet, creatinine, glucose, hemoglobin A1C (HbA1C), total cholesterol (TC), triacylglycerol (TG), low-density lipoprotein cholesterol (LDL-C), high-density lipoprotein cholesterol (HDL-C) (13); (4) medication use, including vasoactive drugs, insulin, antihypertensive drugs, antilipidemic drugs, antiplatelets (17); (5) hospital and ICU admission data, including admission and discharge dates, ICU admission and discharge dates, and death date and time. All measurements utilized in this study were from the initial 24 h post-admission. Specific details and query codes for each metric are available in Supplementary Table S1.

### 2.3 Data definitions

The body mass index (BMI) was calculated as weight (kg) divided by the square of height (m). Patients with diabetes were defined as a history of diabetes or HbA1c > 6.5%. Pre-DM was defined as patients without a history of diabetes but with HbA1c levels between 5.7% and 6.4%. Normoglycemia (NGR) was identified in patients without a history of diabetes or with HbA1c ≤ 5.7% (18).

### 2.4 Exposure variables

A linear regression model between FPG and HbA1c was developed based on all patients included in this study. Based on this, the predicted HbA1c was calculated (predicted HbA1c = 0.015 × FPG + 4.350), and subsequently, the difference between the observed and predicted values of HbA1c was calculated as the HGI (19). The correlation between HGI and HbA1c is shown in Supplementary Figure S1.

### 2.5 Outcome variables

The primary outcomes were mortality rates at 90 and 180 days post-admission, calculated based on the first AMI diagnosis time and the follow-up death dates from MIMIC-IV 2.2.

### 2.6 Statistical analysis

Based on the baseline HGI quartiles, data are expressed as mean (SD) or median (interquartile range) for continuous variables and frequency (percentage) for categorical variables. Differences in HGI levels were assessed using the chi-squared test (for categorical variables), one-way ANOVA (for normally distributed data), and the Kruskal–Wallis H test (for skewed data). The third quartile of HGI served as the reference group. To analyze the association between the HGI and the risk of 90-day and 180-day death, the multiple Cox proportional hazards regression models were utilized to calculate hazard ratios (HRs) and 95% confidence intervals (CIs). Model 1 did not adjust for any covariates. Any covariate altering the resulting estimate by >10% was included in Model 2 as a potential confounder. If the covariate changed the resulting estimate by >10% or had a regression coefficient *P*-value of <0.1, it was considered a potential confounder in Model 3 (20). To prevent multicollinearity, variables with a variance inflation factor (VIF) greater than five were excluded from the models. The Kaplan–Meier survival analysis was also employed to assess endpoint occurrence rates based on different HGI levels.

Likewise, analyses were divided into groups according to the following factors: age, sex, BMI, hypertension, heart failure, stroke, and diabetes. Except for the stratification variable, the adjustment technique was the same as in Model 3. Log-likelihood ratio tests were employed to evaluate interactions between HGI and outcomes between subgroup factors.

Additionally, we employed restricted cubic splines to explore dose–response relationships according to the Akaike information criterion (AIC) minimum criteria. We then calculated HR for HGI and outcomes by running a log-likelihood ratio test for the non-linearity of the smooth curve fit, contrasting the segmented regression model to the single-linear (non-segmented) model while accounting for relevant confounders.

The highest covariate missing rate was the BMI (29.63%). We employed a multiple imputation and chained equation technique in the R MI process to account for the missing data (BMI, WBC, RBC, PLT, and creatinine) to prevent a deterioration in the effectiveness and bias of the statistical analyses owing to the direct exclusion of missing values (20). We performed sensitivity analyses to assess how reliable our results were. Firstly, distributions of variables with missing data are compared to observed complete case data. Secondly, the association between HGI and the risk of 90-day or 180-day mortality was investigated using data before multiple imputations (*n* = 1,358). Thirdly, further adjustments were made to the SIRS and SOFA scores based on clinical data (*n* = 1,360). Finally, recognizing the varying diabetes status among patients, we performed subgroup analyses. R 4.3.0 (http://www.R-project.org) was used for all analyses. A two-sided *P*-value of <0.05 was deemed statistically significant.

## 3 Result

### 3.1 Baseline characteristics of patients with AMI

Data were derived from 1,961 AMI patients (mean age, 66.65 years; 69.10% male), of whom 210 patients experienced a fatal event during the 180-day follow-up period. Baseline data based on HGI quartiles are shown in Table 1. Those with elevated HGI tended to be diabetes patients with increased BMI and HbA1c. However, the proportion of patients with high WBC, cardiogenic shock, and atrial fibrillation was greater in the low HGI group. In addition, we found that glucose, heart failure, and vasoactive drugs use were higher in both the high and low HGI groups than in the median HGI group. Notable differences in BMI, WBC, RBC, PLT, TC, TG, LDL-C, HDL-C, creatinine, glucose, HbA1c, heart failure, cardiogenic shock, diabetes, antihypertensive drugs, antilipidemic drugs, antiplatelets, vasoactive drugs, and 90-day and 180-day mortality were statistically significant across the three patient groups (all *P* < 0.05) (Table 1).

**Table 1:** Baseline characteristics of patients according to quartiles of hemoglobin glycation index.

Hemoglobin glycation index (HGI) quartile					
Characteristics	Q1 (<−0.673)	Q2 (≥−0.673, <−0.281)	Q3 (≥−0.281, <0.234)	Q4 (≥0.234)	*P*-value
Patients, *n*	489	490	490	492	
Age (years)	66.54 (14.07)	66.14 (12.83)	67.33 (13.48)	66.59 (12.44)	0.559
Sex %					0.738
Male	341 (69.73%)	342 (69.80%)	342 (69.80%)	330 (67.07%)	
Female	148 (30.27%)	148 (30.20%)	148 (30.20%)	162 (32.93%)	
BMI kg/m^2^	28.69 (7.15)	28.90 (7.50)	29.11 (8.08)	30.08 (7.43)	0.021
WBC (1,000 cells/ul)	11.82 (5.63)	10.88 (4.61)	10.56 (4.84)	10.14 (4.46)	<0.001
RBC (1,000 cells/ul)	4.15 (0.80)	4.36 (0.74)	4.40 (0.64)	4.30 (0.75)	<0.001
PLT (1,000 cells/ul)	225.84 (72.27)	234.49 (75.61)	240.77 (83.72)	236.39 (80.54)	0.024
TC (mmol/L)	4.48 (1.01)	4.26 (1.03)	4.29 (1.06)	4.25 (1.05)	<0.001
TG (mmol/L)	1.44 (0.96)	1.39 (0.98)	1.36 (0.95)	1.48 (1.01)	<0.001
LDL-C (mmol/L)	2.55 (0.78)	2.51 (0.80)	2.48 (0.81)	2.45 (0.79)	<0.001
HDL-C (mmol/L)	1.10 (0.26)	1.08 (0.24)	1.11 (0.25)	1.07 (0.28)	<0.001
Creatinine (mg/dl)	1.00 (0.80–1.40)	1.00 (0.80–1.20)	1.00 (0.80–1.20)	1.00 (0.80–1.30)	<0.001
Glucose (mg/dl)	155.48 (70.57)	116.02 (33.96)	118.03 (41.03)	153.68 (63.49)	<0.001
HbA1c (%)	5.53 (0.76)	5.68 (0.53)	6.11 (0.66)	8.46 (1.96)	<0.001
Heart failure %	178 (36.40%)	116 (23.67%)	137 (27.96%)	165 (33.54%)	<0.001
Cardiogenic shock %	78 (15.95%)	41 (8.37%)	35 (7.14%)	36 (7.32%)	<0.001
Cardiac arrest %	15 (3.07%)	8 (1.63%)	8 (1.63%)	6 (1.22%)	0.157
Atrial fibrillation %	145 (29.65%)	114 (23.27%)	114 (23.27%)	122 (24.80%)	0.068
Hypertension %	205 (41.92%)	233 (47.55%)	243 (49.59%)	238 (48.37%)	0.078
Diabetes %	123 (25.15%)	80 (16.33%)	150 (30.61%)	417 (84.76%)	<0.001
Antihypertensive drugs %	470 (96.11%)	470 (95.92%)	478 (97.55%)	486 (98.78%)	0.025
Antilipidemic drugs %	437 (89.37%)	468 (95.51%)	477 (97.35%)	472 (95.93%)	<0.001
Antiplatelets %	457 (93.46%)	480 (97.96%)	477 (97.35%)	477 (96.95%)	<0.001
Vasoactive drugs %	201 (41.10%)	154 (31.43%)	144 (29.39%)	202 (41.06%)	<0.001
90-day mortality %	74 (15.13%)	34 (6.94%)	29 (5.92%)	38 (7.72%)	<0.001
180-day mortality %	82 (16.77%)	47 (9.59%)	41 (8.37%)	40 (8.13%)	<0.001

Mean (SD) or median (interquartile range) for continuous variables and as frequencies (percentages) for categorical variables.

BMI, body mass index; WBC, white blood cell; RBC, red blood cell; PLT, platelet; TC, total cholesterol; TG, triacylglycerol; LDL-C, low-density lipoprotein cholesterol; HDL-C, high-density lipoprotein cholesterol.

### 3.2 Associations between HGI and outcomes

Over the 90-day and 180-day follow-up periods post-admission, there were 175 and 210 recorded deaths, respectively. After multivariable adjustment for age, sex, BMI, cardiogenic shock, cardiac arrest, and hypertension, compared with the reference quartile, the first quartile showed a significant association with HGI concerning 90-day and 180-day mortality in Model 2. With further adjustments for potential confounder, the results were consistent in Model 3, multivariable-adjusted HRs (95% CIs) quartiles of across HGI were 1.99 (1.22, 3.08), 1.12 (0.65, 1.94), 1.00 (reference), and 1.26 (0.71, 2.03) (*P* for trend = 0.006) and 1.74 (1.18, 2.43), 1.23 (0.75, 1.76), 1.00 (reference), and 0.93 (0.58, 1.46) (*P* for trend = 0.003), respectively (Table 2).

**Table 2:** Multivariable Cox regression analyses for 180-day and 90-day mortality in patients with acute myocardial infarction.

Outcomes exposure	Model 1 HR (95% CI)	Model 2 HR (95% CI)	Model 3 HR (95% CI)
90-day mortality			
HGI index	0.79 (0.69, 0.91)	0.85 (0.75, 0.98)	0.92 (0.81, 1.04)
HGI index (quartiles)			
Q1	2.72 (1.77, 4.18)***	2.54 (1.65, 3.91)***	1.99 (1.22, 3.08)***
Q2	1.18 (0.72, 1.94)	1.29 (0.78, 2.12)	1.12 (0.65, 1.94)
Q3	Ref	Ref	Ref
Q4	1.31 (0.81, 2.13)	1.46 (0.90, 2.38)	1.26 (0.71, 2.03)
*P* for trend	<0.001	<0.001	0.006
180-day mortality			
HGI index	0.79 (0.70, 0.90)	0.85 (0.75, 0.96)	0.91 (0.81, 1.02)
HGI index (quartiles)			
Q1	2.14 (1.47, 3.12)***	2.03 (1.39, 2.95)***	1.74 (1.18, 2.43)***
Q2	1.16 (0.76, 1.76)	1.27 (0.83, 1.93)	1.23 (0.75, 1.76)
Q3	Ref	Ref	Ref
Q4	0.98 (0.63, 1.51)	1.09 (0.71, 1.70)	0.93 (0.58, 1.46)
P for trend	<0.001	<0.001	0.003

*P < 0.05, **P < 0.01, ***P < 0.001.

BMI, body mass index; WBC, white blood cell; RBC, red blood cell; PLT, platelet; HGI, hemoglobin glycation index; TC, total cholesterol; TG, triacylglycerol; LDL-C, low-density lipoprotein cholesterol; HDL-C, high-density lipoprotein cholesterol.

Model 1: no covariates were adjusted.

Model 2: we only adjusted for age, sex (male, female), BMI, cardiogenic shock (yes, no), cardiac arrest (yes, no), and hypertension (yes, no).

Model 3: we additionally adjusted for WBC, RBC, PLT, TC, TG, LDL-C, HDL-C, creatinine, diabetes (yes, no), heart failure (yes, no), atrial fibrillation (yes, no), insulin (yes, no), antihypertensive drugs (yes, no), antilipidemic drugs (yes, no), antiplatelets (yes, no), vasoactive (yes, no).

**Figure 2:**
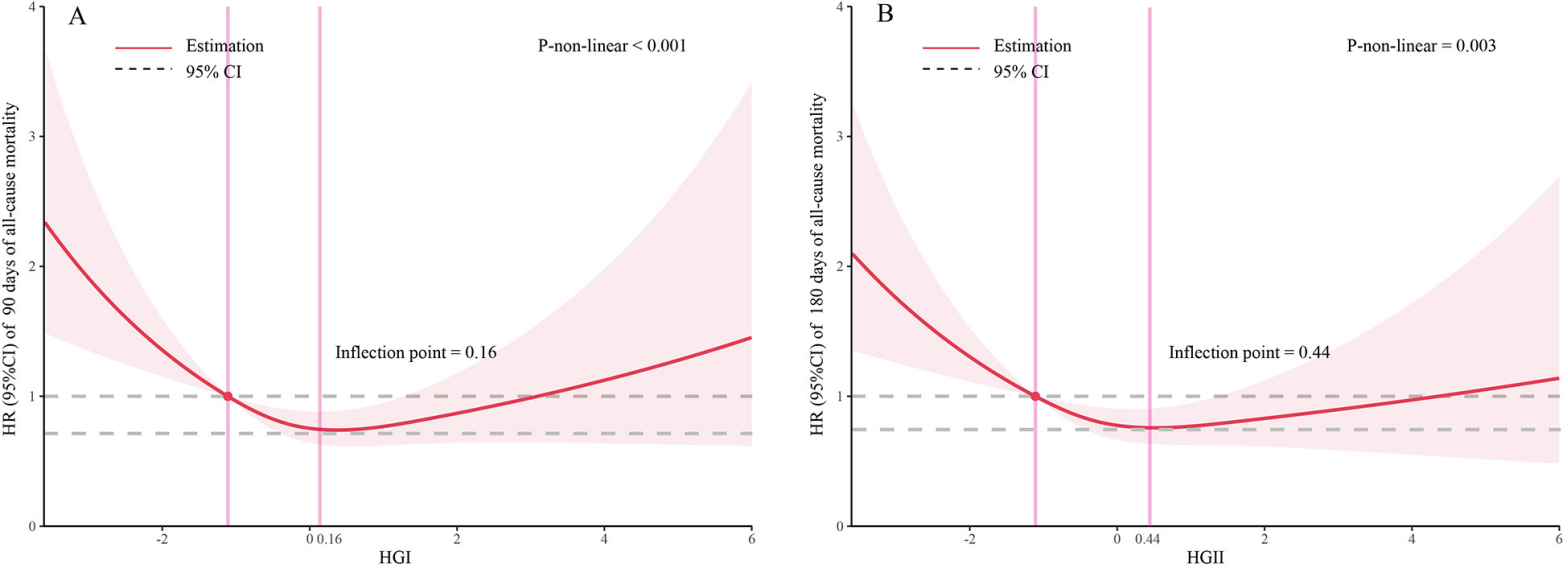
Restricted cubic spline analyses and comparison of the association of HGI with all-cause mortality: **(A)** all-cause death in 90 days, **(B)** all-cause death in 180 days. Heavy central lines represent the estimated adjusted hazard ratios. The 95% confidence interval is represented by the red band. The adjustment strategy is the same as the Model 3.

**Figure 3:**
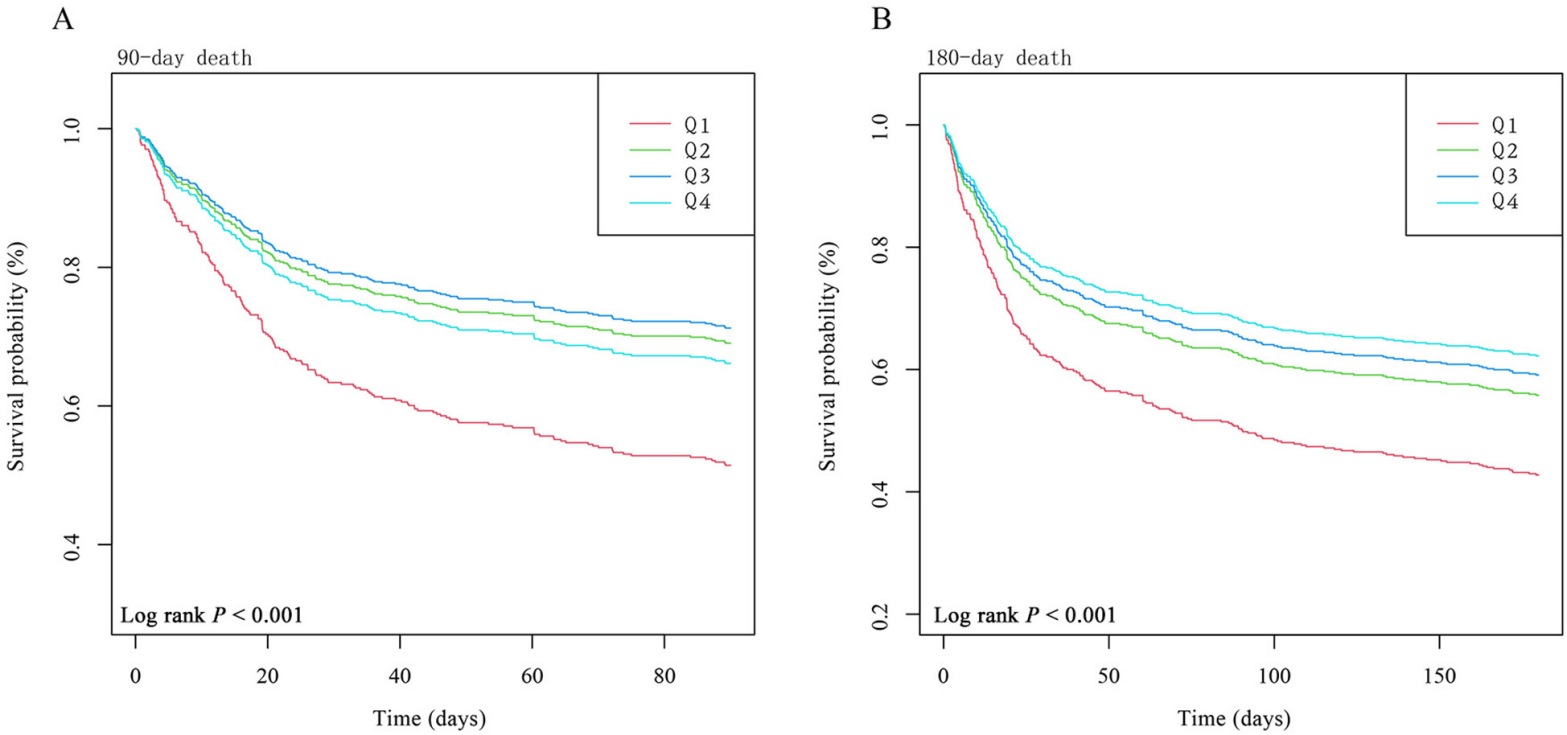
Kaplan–Meier survival analysis curves for all-cause mortality. Footnote: HGI index quartile. Kaplan–Meier curves showing cumulative probability of all-cause mortality according to groups at 90 days **(A)** and 180 days **(B)**. The adjustment strategy is the same as the Model 3.

The dose–response relationship between HGI and the adjusted hazard ratio for 90-day and 180-day mortality in AMI patients was depicted using restricted cubic splines. A L-shaped association between HGI and the 90-day and 180-day mortality rates was observed (Figure 2). Additionally, a combination of Cox proportional hazard models with a two-segmented Cox proportional hazards model was employed to study the non-linear relationship between HGI levels in AMI patients and the mortality rates mentioned above (*P* for log-likelihood ratio <0.05) (Supplementary Table S2). Inflection points were detected at HGI of 0.16 and 0.44. When the HGI is lower than 0.16 and 0.44, for each unit increase in the HGI level, the adjusted HRs for 90-day and 180-day mortality decrease by 28% [HR, 0.72; 95% CI (0.61, 0.85)] and 25% [HR, 0.75; 95% CI (0.64, 0.88)], respectively. We compared the incidence of the outcome between groups using Kaplan–Meier survival analysis curves based on HGI quartiles. The Q1 group had significantly higher mortality rates at 90 and 180 days than the other groups (log-rank *P* < 0.001) (Figure 3). This suggests that low HGI is detrimental to the survival of patients with AMI.

### 3.3 Stratified analysis

Stratification of studies by age, sex, BMI, hypertension, heart failure, cardiogenic shock, and diabetes was conducted to explore the associations with 90-day and 180-day mortality. Consistent results were observed in people over age 65 who were female, with a BMI of 25–30 kg/m^2^, with cardiogenic shock, and without heart failure or diabetes. The model's interaction tests for covariates with HGI were non-significant (*P* for interaction > 0.05) (Supplementary Table S3, S4).

### 3.4 Sensitivity analysis

The characteristics of raw data and data after multiple imputations are presented in Supplementary Table S5. Similar results were observed when we used data before multiple imputations to investigate the association between HGI and the risk of 90-day and 180-day mortality using multivariate Cox regression models (Supplementary Table S6). Similar results were also observed when further adjusting for SIRS and SOFA (Supplementary Table S7). When we conducted a subgroup analysis for individuals with different diabetes statuses, the results were consistent among those with prediabetes (Supplementary Tables S8, S9).

## 4 Discussion

In this study, we used data from the MIMIC-IV database (version 2.2) to reveal an independent association between HGI and 90- and 180-day mortality in patients with a first diagnosis of AMI. This association was particularly evident in patients with prediabetes. In addition, we observed an L-shaped curve relationship between HGI levels and 90- and 180-day mortality in patients diagnosed with AMI. Therefore, HGI may be an independent risk factor for AMI patients. Understanding and HGI levels in patients with AMI may help to improve subsequent health outcomes in these individuals. In clinical practice, linear regression models are needed to calculate HGI in large samples of patients (7, 9, 21, 22).

Several previous studies have shown that HGI is strongly associated with adverse cardiovascular events (23), such as subclinical myocardial injury (SC-MI) (24), prognosis of coronary artery disease (25), and coronary artery calcification (26). One study showed that the proportion of SC-MI participants gradually increased with increasing HGI and that HGI was independently associated with a higher risk of SC-MI in participants without diabetes (24). In a cohort study from China, both low and high HGI were associated with an increased risk of poor outcome in patients with acute coronary syndromes after a median follow-up time of 3 years (13). However, in another cohort study of 1,910 patients with T2DM, low HGI was found to be a possible risk factor for myocardial infarction in patients with coronary artery disease, but the benefit was limited compared with HbA1c (22).

The HGI quantifies the magnitude and direction of the difference between the set of observed and predicted HbA1c outcomes for each patient, and additionally, it is noteworthy that the FPG levels in Q1 were significantly higher than the other groups in our study. Stress hyperglycemia may lead to high FPG followed by low HGI, and stress activates the hypothalamic–pituitary–adrenal axis and the sympathetic–adrenal system, increasing the release of pro-inflammatory cytokines that exacerbate the severity of coronary artery disease in patients with coronary artery disease (27) such as leading to endothelial dysfunction and exacerbating microvascular obstruction, which in turn damages the endothelium of the blood vessels (28). Thus, in the present study, relatively high stress glucose in the low HGI group may have mediated an increased risk of short-term all-cause mortality in AMI patients.

In addition, our findings emphasize the strong association between HGI and mortality, especially in patients with pre-DM but not in those with diabetes. The underlying mechanism for the significantly increased risk of in-hospital death in non-diabetic patients compared with diabetic patients is elusive and may be attributed to several factors. Firstly, our study focused on mortality at 90 and 180 days after admission, representing short-term mortality. Secondly, based on the available evidence, glycation is a complex biological process, and factors affecting intracellular glucose concentration or non-enzymatic hemoglobin glycosylation may also influence the degree of hemoglobin glycation (29). Diabetic patients may exhibit insensitivity to HGI due to long-term adaptation to chronic inflammation and oxidative stress (30). Finally, FPG can be altered to varying degrees in diabetic patients treated with glucose-lowering drugs, with FPG levels being much lower than normal in patients with previous regular insulin therapy (19). They may therefore have a higher HGI. We should consider the potential beneficial outcomes in diabetic patients treated with intensive glucose-lowering therapy or other anti-inflammatory drugs despite adjusting insulin use (31). Based on stratified analyses, we observed that low HGI was associated with increased mortality in patients with AMI, especially in patients older than 65 years, with a BMI of 25–30 kg/m^2^, and without heart failure and diabetes. Therefore, it can be used as a potential indicator for risk stratification of mortality in such patients and should be given extra attention in clinical practice.

Our study has several limitations. First, as a single-center study with a limited sample size, even though multivariate adjustment and subgroup analysis were performed, we may not have extracted the full clinical diagnostic information and sociodemographic indicators of the patients, and potential bias due to residual confounders may persist. Second, the association between HGI and adverse outcomes other than all-cause mortality was not considered in this study. Third, while we excluded CKD stage 5 patients, data on earlier CKD stages and diabetic microvascular complications (e.g., retinopathy, neuropathy) were unavailable in the MIMIC-IV database. These factors may influence mortality risk and should be incorporated in future studies. Fourth, medication compliance after hospital discharge was not assessed due to the lack of longitudinal outpatient data in MIMIC-IV. While in-hospital medication use was adjusted for in our models, post-discharge adherence to therapies (e.g., statins, antiplatelets) may confound long-term mortality risk and warrants further investigation, and future studies need to further explore the interaction between HGI and different treatment modalities. Finally, in our study, HGI was calculated based on the study population and could not be generalized to other populations. We believe that regression models should be built based on data retrieved from various large databases to calculate HGI in various populations.

## 5 Conclusion

Using patient data retrieved from the MIMIC-IV database, we found a non-linear relationship between HGI and all-cause mortality in patients with AMI. Our findings emphasize that low HGI is associated with increased mortality. We suggest that HGI is a good indicator of poor prognosis in patients with AMI, especially in prediabetic patients, and that this could be used as a potential indicator for risk stratification of mortality in such patients. Patients with AMI who have a low HGI should receive extra attention during hospitalization.

## Data Availability

The original contributions presented in the study are included in the article/Supplementary Material, further inquiries can be directed to the corresponding author.
