# Supplementary material for: Association between different hemoglobin glycation index and prognosis in patients with a first diagnosis of acute myocardial infarction: a retrospective study based on the MIMIC-IV database
Source: Front Cardiovasc Med. 2025 May 26;12:1447420. doi: 10.3389/fcvm.2025.1447420 (PMC12146337; doi:10.3389/fcvm.2025.1447420)
Supplement: Supplementary file 1 [file Datasheet1.pdf]

Supplementary Materials

Table S1. Variables query code in MIMIC-IV.

Table S2. Threshold effect analysis of HGI index on 90-day and 180-day mortality in acute myocardial infarction patients. The adjustment strategy is the same as the Model 3.

Table S3. Subgroup analysis with 90-day mortality as the outcome event. With the exception of the stratified variable itself, the adjustment approach is the same as for Model 3 in the manuscript.

Table S4. Subgroup analysis with 180-day mortality as the outcome event. With the exception of the stratified variable itself, the adjustment approach is the same as for Model 3 in the manuscript.

Table S5. Distributions of variables with missing data comparing observed complete case data to results from pooling the datasets with imputed variables from multiple imputation.

Table S6. Multivariable Cox regression analyses for 90-day and 180-day mortality in patients with acute myocardial infarction using raw data.

Table S7. Multivariable Cox regression analyses for 90-day and 180-day mortality in patients with acute myocardial infarction. We additionally adjusted for SIRS (systemic inflammatory response) and SOFA (sequential organ failure assessment).

Table S8. Multivariable Cox regression analyses for 90-day mortality in patients with acute myocardial infarction and different diabetes mellitus.

Table S9. Multivariable Cox regression analyses for 180-day mortality in patients with acute myocardial infarction and different diabetes mellitus.

.

Figure S1. The correlation between HGI and HbA1c.

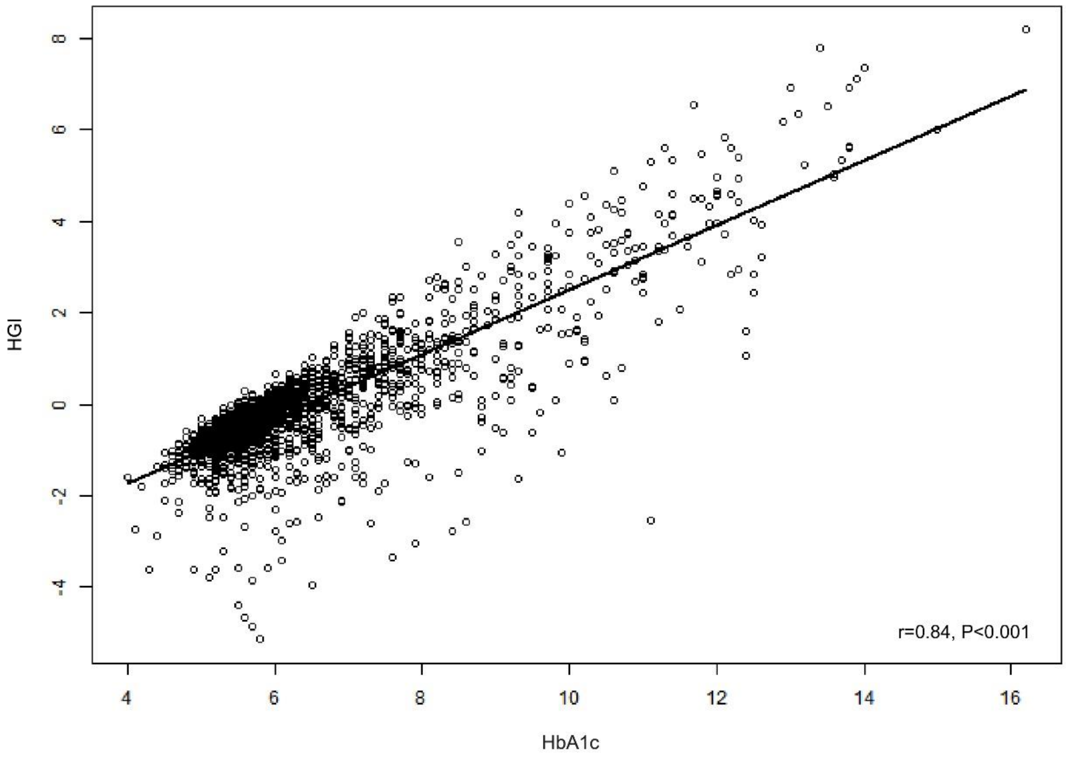

Supplementary Table 1. Variables query code in MIMIC-IV

Variables	Query code
**Acute myocardial infarction**	41001, 41002, 41011, 41012, 41021, 41022, 41031, 41041, 41042, 41051, 41081, 41082, 41091, 41092, I2101, I2102, I2109, I2111, I2119, I2121, I2129, I213, I214, I219, I21A1, I21A9, I222
**Hypertension**	4010, 4011, 4019, 40591, I10
**Cardiogenic shock**	78551, R570
**Atrial fibrillation**	42731; I480; I481; I4811; I4819; I482; I4820; I4821; I4891
**Chronic kidney disease stage 5**	40311; 40391; 40413; 40493; I120; I1311; I132
**Cardiac arrest**	4275, I46
**Heart failure**	
acute heart failure	42821, 42823, 42831, 42833, 42841, 42843, I5021, I5023, I5031, I5033, I5043,
chronic heart failure	I5043,42822;42832;42842;42843;I5022;I5032;I5042; I50812;I50813;I5084
**Diabetes**	E10, E11, E13
**Malignant tumor**	C220; D0462; 2330; 17331; Z85821; D0511; C44310
**Vasoactive drugs**	
norepinephrine	221906
dopamine	221662
epinephrine	221289
deoxyadrenaline	221749; 229630; 229632
dobutamine	221653
**Antihypertensive drugs**	
angiotensin converting enzyme inhibitors	BENA10; BENA20; BENA5; CAPT1; CAPT125; CAPT25; EMLA2.5; ENAL/P2.5; ENAL/P5; ENAL1.25I; ENAL10; ENAL25I; ENAL25IVPB; ENAL5; FOSI10; FOSI20; FOSI40; LISI10; LISI2.5; LISI20; LISI5; LOTR5/20; MOEX15; MOEX7.5; QUIN20; QUIN5; RAMI/PLACIND; RAMI125; RAMI5; TRAN1; TRAN2
angiotensin II receptor blockers	CAND16; CAND4; COZA25; COZAA50; ENTR24/26; ENTR49/51; ENTR97/103; IRBE150; IRBE300; LCZ696/50; LCZ696/P50; OLME20; TELM20; VALS160; VALS40; VALS80
calcium-channel blockers	ADAL30; ADAL60; ADAL90; AMLO25; AMLO5; CLEV25I; EG-1962/P; FELO2.5; ISRA25; LOTR5/20; NICA2.5I; NICA20; NICA30; NICA40PM; NIFE10; NIMO30; NIMO30/P NIMO30/PS; NIMO30L; SYRINGEPD
clonidine	CATA1; CATA2; CLON1I; CLON5I
hydralazine	HYDZ10; HYDZ20I; HYDZ25; HYDZ50
diuretic	FURO100DES; FURO100I; FURO100PB; FURO100S; FURO10L; FURO10S; FURO1MCGS; FURO1S; FURO20; FURO20I; FURO40; FURO40/20I; FURO40HIND; FURO40I; FURO40ILF; FURO40L FURO80,SPIR100; SPIR12.5HT; SPIR25,BISO5; CHL500I; CHL500PB; CHLO250; DYAZ1; HCTZ12.5; HCTZ25; HCTZ50; MAXZ25
beta blockers	BISO5; METO12.5HT; METO1L; METO25; METO37.5; METO50; METO5I; METOSUSP; METOSUSP10L; TOPR100; TOPR25; TOPR50
**Antilipidemic drugs**	ATOR/40/P; ATOR/PLACIND; ATOR10; ATOR20; ATOR40; ATOR80; ATORSTUDY; FLUV20; IMIP500I; LIPI40BN; LOVA20; MICO2P; MYC30O; MYCO2C; NYST100P; NYST100V; NYST500T; NYST5L; NYST60L; NYST60LSM; NYSTC; NYSTO; PENT10I; PENT10SYR; PENTO10I; PRAV10; PRAV10L; PRAV20; PRAV40; ROSU20; ROSU20INV; ROSU5; SIMV10; SIMV40; SIMVIND,FENO145; FENO48
**Antiplatelets**	AGGRCAP; ASA300R; ASA325; ASA600R; ASA81; ASA81EC; ASAB325; ASAEC325; ASPI20.25QT; ASPI325INDP; ASPI40.5HT; ASPI81/PLB; ASPI81INDP; ASPIDESEN; FIOR,CLO025L; CLOP.25L; CLOP0.5L; CLOP01L; CLOP05L; CLOP15L; CLOP1L; CLOP2.5L; CLOP300; CLOP5L; CLOP7.5L; CLOP75; CLOP75/P; CLOP75/PIND; CLOPIND; NACLFLUSH
**Insulin**	1740，27413，44340，47172，47780
**Weight (Kg)**	226512
**RBC (K/μL)**	51279
**Platelet (K/μL)**	51265
**WBC (K/μL)**	51755

**Creatinine (mg/dL)**	52024
**Glucose (mg/dL)**	220621
**HbA1c (%)**	50852

Table S2. Threshold effect analysis of HGI index on 90-day and 180-day mortality in acute myocardial infarction patients. The adjustment strategy is the same as the Model 3.

	Adjusted HR (95% CI), P-value
**90-day mortality**	
Standard linear regression	0.92 (0.81, 1.04) 0.173
Fitting model by two-piecewise linear regression	
Inflection point	0.16
HGI index < 0.16	0.72 (0.61, 0.85) <0.001
HGI index > 0.16	1.15 (0.99, 1.33) 0.068
P for the Log-likelihood ratio	<0.001
**180-day mortality**	
Standard linear regression	0.91 (0.81, 1.02) 0.103
Fitting model by two-piecewise linear regression	
Inflection point	0.44
HGI index < 0.44	0.75 (0.64, 0.88) <0.001
HGI index > 0.44	1.12 (0.96, 1.31) 0.136
P for the Log-likelihood ratio	0.003

Table S3. Subgroup analysis with 90-day mortality as the outcome event. With the exception of the stratified variable itself, the adjustment approach is the same as for Model 3 in the manuscript.

Subgroups	Hemoglobin glycation index (quartiles)				
	Q1	Q2	Q3	Q4	P for interaction
Age, years					0.86
< 65	1.72 (0.61, 4.79)	1.21 (0.39, 3.74)	Ref	1.38 (0.46, 4.16)	
≥ 65	**1.96 (1.18, 3.25)**	1.23 (0.69, 2.19)	Ref	1.02 (0.56, 1.86)	
Sex					0.81
Male	**1.92 (1.07, 3.44)**	1.32 (0.70, 2.48)	Ref	1.17 (0.61, 2.24)	
Female	**2.18 (1.05, 4.53)**	0.94 (0.40, 2.26)	Ref	1.23 (0.51, 2.93)	
BMI, kg/m2					0.94
< 25	1.66 (0.75, 3.64)	1.15 (0.49, 2.71)	Ref	1.36 (0.54, 3.42)	
≥ 25, <30	**2.97 (1.33, 6.66)**	1.58 (0.56, 4.42)	Ref	1.40 (0.53, 3.67)	
≥ 30	1.49 (0.64, 3.48)	1.16 (0.48, 2.79)	Ref	1.11 (0.43, 2.91)	
Hypertension					0.06
No	**2.30 (1.28, 4.15)**	1.02 (0.51, 2.05)	Ref	1.69 (0.89, 3.23)	
Yes	1.77 (0.86, 3.62)	1.59 (0.73, 3.45)	Ref	0.44 (0.15, 1.34)	
Heart failure					0.15
No	**1.95 (1.05, 3.63)**	1.09 (0.55, 2.16)	Ref	0.67 (0.31, 1.43)	
Yes	**2.14 (1.08, 4.23)**	1.35 (0.61, 2.99)	Ref	1.99 (0.94, 4.19)	
Stroke					0.09
No	1.60 (0.96, 2.68)	1.05 (0.60, 1.86)	Ref	0.92 (0.52, 1.64)	
Yes	**5.61 (1.86, 16.92)**	2.07 (0.57, 7.45)	Ref	**3.70 (1.00, 13.63)**	
Diabetes					0.94
No	**2.54 (1.41, 4.59)**	1.38 (0.70, 2.71)	Ref	1.39 (0.53, 3.65)	
Yes	1.99 (0.96, 4.15)	1.16 (0.49, 2.78)	Ref	1.10 (0.55, 2.20)	

Table S4. Subgroup analysis with 180-day mortality as the outcome event. With the exception of the stratified variable itself, the adjustment approach is the same as for Model 3 in the manuscript.

Subgroups	Hemoglobin glycation index (quartiles)				
	Q1	Q2	Q3	Q4	P for interaction
Age, years					0.73
< 65	1.74 (0.68, 4.46)	1.37 (0.50, 3.75)	Ref	1.24 (0.44, 3.46)	
≥ 65	**1.55 (1.00, 2.40)**	1.17 (0.72, 1.89)	Ref	0.72 (0.42, 1.23)	
Sex					0.49
Male	1.59 (0.95, 2.66)	1.39 (0.81, 2.37)	Ref	0.91 (0.51, 1.62)	
Female	1.76 (0.93, 3.30)	0.82 (0.39, 1.72)	Ref	0.85 (0.39, 1.88)	
BMI, kg/m2					0.90
< 25	1.49 (0.76, 2.91)	1.08 (0.53, 2.23)	Ref	1.03 (0.45, 2.36)	
≥ 25, <30	**2.33 (1.12, 4.81)**	2.08 (0.89, 4.83)	Ref	1.18 (0.49, 2.80)	
≥ 30	1.29 (0.62, 2.68)	1.08 (0.51, 2.32)	Ref	0.68 (0.29, 1.61)	
Hypertension					0.07
No	1.63 (0.99, 2.66)	0.97 (0.55, 1.69)	Ref	1.13 (0.65, 1.96)	
Yes	1.79 (0.92, 3.49)	1.68 (0.84, 3.38)	Ref	0.32 (0.11, 0.91)	
Heart failure					0.09
No	**1.82 (1.05, 3.16)**	1.34 (0.76, 2.37)	Ref	0.57 (0.29, 1.14)	
Yes	1.45 (0.81, 2.59)	0.99 (0.49, 1.98)	Ref	1.26 (0.65, 2.43)	
Stroke					0.06
No	1.43 (0.91, 2.24)	1.14 (0.71, 1.84)	Ref	0.73 (0.43, 1.23)	
Yes	**4.35 (1.64, 11.51)**	1.80 (0.58, 5.65)	Ref	2.60 (0.79, 8.55)	
Diabetes					0.95
No	**2.05 (1.22, 3.42)**	1.31 (0.75, 2.31)	Ref	1.00 (0.40, 2.50)	
Yes	1.62 (0.85, 3.11)	1.24 (0.60, 2.59)	Ref	0.84 (0.46, 1.55)	

Table S5. Distributions of variables with missing data comparing observed complete case data to results from pooling the datasets with imputed variables from multiple imputation. Abbreviations: BMI: body mass index; WBC: white blood cell; RBC: red blood cell; PLT: platelet.

	Level/ Unit	Number (%) with missing data	Complete case	Multiple imputation	P-value
BMI kg/m2	Mean (SD)	29.63	29.69 (6.62)	29.20 (7.56)	0.209
WBC (1000 cells/uL)	Mean (SD)	1.02	10.78 (4.66)	10.85 (4.94)	0.924
RBC (1000 cells/uL)	Mean (SD)	0.31	4.31 (0.73)	4.30 (0.74)	0.877
PLT (1000 cells/uL)	Mean (SD)	2.86	235.16 (78.10)	234.38 (78.29)	0.738
Creatinine (mg/dL)	Median (interquartile range)	0.92	1.00 (0.80-1.20)	1.00 (0.80-1.20)	0.914

Table S6. Multivariable Cox regression analyses for 90-day and 180-day mortality in patients with acute myocardial infarction using raw data. Model: we adjusted for age, sex (male, female), BMI, WBC, RBC, PLT, TC, TG, LDL-C, HDL-C, creatinine, heart failure (yes, no), atrial fibrillation (yes, no), diabetes (yes, no), insulin (yes, no), antihypertensive drugs (yes, no), antilipidemic drugs (yes, no), antiplatelets (yes, no), vasoactive (yes, no). (n=1358) Abbreviations: BMI: body mass index; WBC: white blood cell; RBC: red blood cell; PLT: platelet; TC: total cholesterol; TG: triacylglycerol; LDL-C: low-density lipoprotein cholesterol; HDL-C: high-density lipoprotein cholesterol.

.

	Hemoglobin glycation index (HGI) quartile				
	Q1	Q2	Q3	Q4	P for trend
**90-day mortality**					
Model HR,95%CI	1.85 (1.05, 3.27)*	1.15 (0.61, 2.18)	ref	1.30 (0.69, 2.46)	0.091
**180-day mortality**					
Model HR,95%CI	1.61 (1.04, 2.66)*	1.08 (0.62, 1.87)	ref	1.01 (0.57, 1.79)	0.041

Table S7. Multivariable Cox regression analyses for 90-day and 180-day mortality in patients with acute myocardial infarction. We additionally adjusted for SIRS (systemic inflammatory response) and SOFA (sequential organ failure assessment). Model: we adjusted for age, sex (male, female), BMI, WBC, RBC, PLT, TC, TG, LDL-C, HDL-C, creatinine, heart failure (yes, no), atrial fibrillation (yes, no), diabetes (yes, no), insulin (yes, no), antihypertensive drugs (yes, no), antilipidemic drugs (yes, no), antiplatelets (yes, no), vasoactive (yes, no), SIRS and SOFA. (n=1360) Abbreviations: BMI: body mass index; WBC: white blood cell; RBC: red blood cell; PLT: platelet; TC: total cholesterol; TG: triacylglycerol; LDL-C: low-density lipoprotein cholesterol; HDL-C: high-density lipoprotein cholesterol.

.

	Hemoglobin glycation index (HGI) quartile				
	Q1	Q2	Q3	Q4	P for trend
**90-day mortality**					
Model HR,95%CI	1.79 (1.08, 2.95)*	1.14 (0.64, 2.04)	ref	1.12 (0.63, 2.00)	0.020
**180-day mortality**					
Model HR,95%CI	1.63 (1.04, 2.57)*	1.22 (0.73, 2.03)	ref	0.91 (0.54, 1.55)	0.005

Table S8. Multivariable Cox regression analyses for 90-day mortality in patients with acute myocardial infarction and different diabetes mellitus.

P-value *P<0.05 **P<0.01 ***P<0.001.

Abbreviations: BMI: body mass index; WBC: white blood cell; RBC: red blood cell; PLT: platelet; TC: total cholesterol; TG: triacylglycerol; LDL-C: low-density lipoprotein cholesterol; HDL-C: high-density lipoprotein cholesterol.

Model 1: no covariates were adjusted.

Model 2: we only adjusted for age, sex (male, female), BMI, cardiogenic shock (yes, no), cardiac arrest (yes, no), hypertension (yes, no).

Model 3: we additionally adjusted for WBC, RBC, PLT, TC, TG, LDL-C, HDL-C, creatinine, heart failure (yes, no), atrial fibrillation (yes, no), insulin (yes, no), antihypertensive drugs (yes, no), antilipidemic drugs (yes, no), antiplatelets (yes, no), vasoactive (yes, no).

Diabetes status	Model 1<br>HR,95%CI	Model 2<br>HR,95%CI	Model 3<br>HR,95%CI
NGR			
HGI index (quartiles)			
Q1	2.24 (0.94, 5.32)	1.86 (0.78, 4.47)	1.68 (0.66, 4.26)
Q2	0.90 (0.35, 2.32)	0.98 (0.38, 2.54)	1.16 (0.43, 3.17)
Q3	Ref	Ref	Ref
Q4	0.00 (0.00, Inf)	0.00 (0.00, Inf)	0.00 (0.00, Inf)
P for trend	0.005	0.043	0.162
Pre-DM			
HGI index (quartiles)			
Q1	9.43 (4.03, 22.06) ***	8.61 (3.54, 20.92) ***	8.30 (2.91, 23.68) ***
Q2	1.96 (0.73, 5.25)	1.77 (0.64, 4.88)	1.75 (0.54, 5.66)
Q3	Ref	Ref	Ref
Q4	1.14 (0.25, 5.28)	1.08 (0.23, 5.02)	1.06 (0.20, 5.54)
P for trend	<0.001	<0.001	<0.001
DM			
HGI index (quartiles)			
Q1	2.47 (1.28, 4.74) **	2.07 (1.06, 4.03) *	1.73 (0.87, 3.45)
Q2	1.77 (0.82, 3.83)	1.63 (0.75, 3.54)	0.95 (0.41, 2.20)
Q3	Ref	Ref	Ref
Q4	0.91 (0.49, 1.69)	1.01 (0.54, 1.88)	0.94 (0.50, 1.77)
P for trend	<0.001	0.005	0.051

Table S9. Multivariable Cox regression analyses for 180-day mortality in patients with acute myocardial infarction and different diabetes mellitus.

P-value *P<0.05 **P<0.01 ***P<0.001.

Abbreviations: BMI: body mass index; WBC: white blood cell; RBC: red blood cell; PLT: platelet.

Model 1: no covariates were adjusted.

Model 2: we only adjusted for age, sex (male, female), BMI, cardiogenic shock (yes, no), cardiac arrest (yes, no), hypertension (yes, no).

Model 3: we additionally adjusted for WBC, RBC, PLT, creatinine, heart failure (yes, no), atrial fibrillation (yes, no), insulin (yes, no), antihypertensive drugs (yes, no), antilipidemic drugs (yes, no), antiplatelets (yes, no), vasoactive (yes, no).

Diabetes status	Model 1<br>HR,95%CI	Model 2<br>HR,95%CI	Model 3<br>HR,95%CI
NGR			
HGI index (quartiles)			
Q1	1.71 (0.83, 3.51)	1.44 (0.69, 2.99)	1.33 (0.60, 2.93)
Q2	0.80 (0.36, 1.75)	0.87 (0.40, 1.92)	1.04 (0.45, 2.42)
Q3	Ref	Ref	Ref
Q4	0.00 (0.00, Inf)	0.00 (0.00, Inf)	0.00 (0.00, Inf)
P for trend	0.019	0.117	0.335
Pre-DM			
HGI index (quartiles)			
Q1	7.28 (3.42, 15.49) ***	6.95 (3.15, 15.36) ***	6.84 (2.86, 16.34) ***
Q2	2.16 (0.97, 4.82)	2.16 (0.95, 4.92)	1.87 (0.74, 4.69)
Q3	Ref	Ref	Ref
Q4	0.79 (0.18, 3.51)	0.74 (0.17, 3.29)	0.74 (0.16, 3.47)
P for trend	<0.001	<0.001	<0.001
DM			
HGI index (quartiles)			
Q1	1.98 (1.10, 3.56) *	1.74 (0.95, 3.17)	1.47 (0.79, 2.73)
Q2	1.75 (0.90, 3.41)	1.65 (0.85, 3.23)	1.05 (0.51, 2.15)
Q3	Ref	Ref	Ref
Q4	0.71 (0.41, 1.23)	0.79 (0.45, 1.39)	0.75 (0.43, 1.33)
P for trend	<0.001	<0.001	0.014
